# Editorial

**Published:** 1851-01

**Authors:** 


### THE MEDICAL EXAMINER.

PHILADELPHIA, JANUARY, 1851.

### TO OUR READERS.

In presenting our readers with the first number of the fourteenth volume of the Medical Examiner, we take great pleasure in announcing that hereafter the editorial department will have the valuable assistance of Dr. John B. Biddle, the originator and first editor of this journal. To most of our readers he is well known, especially to those who have been recipients of the Examiner from its earliest date. Under his care it was born and grew to a goodly stature, and he now returns to it once more with the same zeal that actuated him in its foundation. We congratulate ourselves as well as our readers on this change, being well satisfied that it will work to the advantage of all concerned.

We shall aim, hereafter, to devote an increase of space and attention to our clinical department. This journal is, we believe, the only one in the country, not of very recent date, that has ever systematically attempted to present reports of cliniques, and of hospital and clinical lectures, and we believe that it has always proved an agreeable and interesting feature to our readers. Our arrangements for the future will place at our disposal regular reports of the proceedings of the schools and hospitals of Philadelphia in this particular. It is not our intention, however, to overburthen our columns with a fatiguing detail of trivial and every-day cases, but we shall endeavor to make such a selection from the ample supply of materials, which the metropolis of American medicine is constantly offering, as to present a faithful reflection of its clinical practice, wherever novelty and importance of treatment and cases may claim notice.

It will be found that the cliniques of the two large schools furnish no inconsiderable proportion of our reports. We are aware that, in some quarters, these cliniques have been in disfavor, from an impression that they are designed to take the place of genuine hospital instruction, for which they are certainly not to be considered a substitute. It should be noticed, however, by those who have been led to condemn this method of instruction, that confessedly inferior as it is to the daily minute observation of disease, which is only to be practised by the bedside in the wards of a well regulated hospital, it is really hardly different in character from the *am-*

*phitheatrical* lectures delivered in these institutions. True clinical instruction can be given only to classes of some half dozen or dozen at the bedside, a plan everywhere incompatible with the size of the winter classes here. And when it is borne in mind that a narrow feeling on the part of the managers of our Alms House has closed its fine hospital as a means of study, we think it is unfair to undervalue the college cliniques, where a vast and interesting mass of disease is collected and turned to account, which, but for them, would for the most part be lost to science. In an ethical point of view, the cliniques are perhaps assailable as a source of indiscriminate gratuitous medical service. But we have at present only to do with their scientific and educational bearing.

The original, bibliographical, and foreign departments of the Examiner will claim no less of the editors' attention than heretofore, and they will endeavor still to maintain its reputation for candor and impartiality; sparing no pains to make it in every respect a "Record of Medical Science," worthy of the highly intelligent and respectable list of readers who have so long honored it with their support.

---

### ON THE USE OF TOBACCO.

We have received a communication from Dr. P. C. SUTPHIN, of Bedford county, Virginia, on the "Influence of Tobacco on the Human System," which we regret that the pressure on our columns at present, obliges us to defer publishing in extenso. Dr. SUTPHIN'S attention was led to the topic by an article, which appeared in the Examiner for October last, on the pernicious effects arising from habitual indulgence in the use of tobacco. As the result of considerable experience in the tobacco-growing region of Virginia, where its use is almost universal, "and generally to excess," Dr. SUTPHIN is of opinion that it has no appreciable influence in the development of diseases of the heart or stomach. "It is but seldom we are called upon," he says, "to treat heart diseases, and when we are, we can generally trace their origin to an attack of rheumatism, or some other cause distinct from tobacco. Dyspepsia and other diseases, enumerated in the article which appeared in the October number of the Examiner, are not more common amongst those who use tobacco, than amongst those who do not. Any unprejudiced physician acquainted with these facts, will corroborate the statements made here. Dyspepsia and heart diseases are almost unknown among our negroes, who use a great quantity of tobacco. I have known vast numbers of negroes to chew and smoke almost incessantly, and nevertheless enjoy the greatest possible health."

Testimony like this—from so respectable and well-informed a quarter—certainly goes far to shake the belief, which we take to be general, in the injurious tendency of constant and excessive indulgence in tobacco. "When employed in excess," says Dr. Wood, in the U. S. Dispensatory, "it enfeebles the digestive powers, produces emaciation and general debility, and lays the foundation of serious disorders of the nervous system," and Dr. Chapman "has met with several instances of mental disorder closely resembling delirium tremens, which resulted from its abuse, and which subsided in a few days after it had been abandoned." This is the impression generally of writers on the materia medica. That an article, the effects of which on persons unaccustomed to its use, are so powerful—espcially *on the functions of the stomach and heart*—should find in the human system an adaptability to its action, entirely counteracting its original noxious influence, is not a little remarkable, particularly with our experience of the results of the abuse of opium and alcoholic stimuli.

We must allow, however, that the facts elicited from our correspondent, deserve great weight—not only from his necessarily extended familiarity with the effects of the use of tobacco, but also from his favorable position for observing them, independently of the many causes of gastric and cardiac disorder, which are to be found in large cities. For it may be, that tobacco has been unjustly charged with consequences pernicious to the health, which are really, in the main, traceable to the mental excitement and sedentary habits of town life. We should be glad to hear from others of our readers in the tobacco districts, and, indeed, from any who may have positive facts to communicate on the subject.

---

The length of the original communications in this number, with the mass of clinical matter which has accumulated on our hands, has obliged us somewhat to encroach upon the space usually allotted to our 'Record of Medical Science.' We shall not, however, allow this interesting department of the journal to be permanently abridged—but shall aim to present hereafter, each month, to our readers, a carefully collated and full retrospect of the improvements and discoveries in the different branches of medicine.

---

### MONUMENT TO JENNER.

We learn with pleasure from the British journals, that a bronze statue of Jenner is to be placed in the open air, in some situation in London. Mr. Marshall, the eminent sculptor, who has received the original bust of Jenner from Dr. Baron, is going on with a cast of the intended monument, which he expects to finish, so as to place it in the great exhibition of 1851; and he hopes to complete the work in bronze in 1852.

### TO THE MEDICAL PROFESSION.

The undersigned, Chairman of the Standing Committee on *Practical Medicine*, appointed by the American Medical Association, May, 1850, respectfully solicits the co-operation of the members of the Medical Profession, in furnishing materials for the Annual Report in May, 1851. The duty of this Committee, as defined by the Constitution of the Association, is to "prepare an Annual Report on the more important improvements effected in this country in the management of individual diseases; and on the progress of Epidemics; referring, as occasion requires, to medical topography, and to the character of prevailing diseases in special localities, or in the United States generally, during the term of their service." In order to fulfil the objects thus expressed, the requisite data must be supplied by medical practitioners in different sections of the Union. This is more particularly true with reference to the "*progress of epidemics*," and "*the character of prevailing diseases in special localities.*" Communications, therefore, are particularly desired from persons residing in places in which Epidemics have prevailed, or in which prevailing diseases have been marked by special characters during the present year. Epidemic Cholera and Dysentery are known to have prevailed more or less extensively in different parts of the country during the past summer. Facts bearing upon the features peculiar to the present season, the production, diffusion, mortality, treatment, etc., of these diseases will be acceptable. It is requested that communications upon these or any of the subjects coming under the cognizance of the Committee, be transmitted to the undersigned, by the first of March, 1851.

All contributions with which the Committee may be favored, will receive due attention and acknowledgment.

Austin Flint.

*Buffalo, New York, Nov.*, 1850.

Editors of Medical Journals will confer a favor by copying the above.

---

